# Utilization and Impact of Peer-Support Programs on Police Officers’ Mental Health

**DOI:** 10.3389/fpsyg.2020.01686

**Published:** 2020-07-14

**Authors:** Beth Milliard

**Affiliations:** ^1^York Regional Police, Aurora, ON, Canada; ^2^Criminal Justice, Walden University, Minneapolis, MN, United States

**Keywords:** police officer, peer support (PS), mental health, stigma, policy, provincial standard

## Abstract

Police officer suicide rates hit an all-time high in the province of Ontario, Canada, in 2018. Sadly, this statistic is somewhat unsurprising, as research has shown that police officers suffer from higher rates of mental health disorder diagnoses compared to the general public. One key reason for the elevated levels of suicide and other mental health issues among police officers is believed to stem from the stigma associated with seeking help. In an attempt to address these serious issues, Ontario’s police services have begun to create internal peer support programs as a way of supporting their members. The present research explores the experiences of police officers serving as peer-support team members, particularly with regards to the impacts of peer support. In addition, this research also examines the importance of discussing shared experiences regarding a lack of standardized procedures for the administration and implementation of peer support in relation to the Policy Feedback Theory. The Policy Feedback Theory (PFT) posits that, when a policy becomes established and resources are devoted to programs, it helps structure current activity. This study utilized a phenomenological, qualitative approach, with data collection consisting of face-to-face interviews with nine police officers serving on the York Regional Police’s peer-support team. The findings revealed that peer support is more than just a “conversation”; rather, it suggests to contribute to enhancing mental health literacy among police officers, and it significantly contributes to stigma reduction. The findings also revealed that internal policy demonstrated an organizational commitment to mental health and peer-support, and that a provincial standard is necessary to ensure best practices and risk management in the creation and maintenance of peer-support programs.

## Introduction

Mental health issues among police personnel has been a particular topic of concern for police services in Ontario, Canada, since 2015 (Mental Health Commission of Canada and Public Services Health and Safety Association, 2015). Typically, stress levels and well-being among police officers have been associated with various physiological factors, such as lack of exercise, high blood pressure, and elevated cortisol levels due to shift work (Jensen et al., 2016), as well as other factors inherent to a high-intensity profession where individuals sometimes experience low-levels of control (Garbarino and Magnavita, 2015). As a result, the majority of research on stress and wellness among police officers has primarily focused on these physiological factors and the importance of health and fitness programs (DeNysschen et al., 2018). However, increased rates of post-traumatic stress disorder (PTSD) and other mental health issues among law enforcement has led researchers to look at other stressors that are inherent in the policing profession. For example, the Carleton et al. (2018) study of Canadian public safety personnel (PSP) found that first responders have increased symptoms of mental health disorders compared to the general public. Stanley et al. (2016) have identified a number of factors that may explain this disparity in the prevalence of mental health issues, including: occupational hazards and exposures to traumatic incidents, shift work, stigma associated with reaching out for mental health support, high-risk roles, and overall lack of mental health resources. In addition to PTSD, other mental health issues that present among first responders are major depressive disorder, panic disorder, generalized anxiety disorder, social anxiety disorder, and vulnerability to alcohol use disorder (Carleton et al., 2018). As a result, the focus of research is now leaning toward the effectiveness of proactive mental health programs and initiatives in the policing community which focus around stigma reduction (Smid et al., 2017; Stuart, 2017).

One such initiative that has become prevalent among police services in Ontario is the creation of peer-support teams. Peer-support programs provide police officers with an opportunity to share their experiences with other officers, which is important because fellow officers are perhaps best able to relate to their colleagues’ experiences in the line of duty. Peer-support has been widely accepted as a confidential outlet for police officers to speak to other police officers free from judgment and the sharing of private information. Promoting the sense that police officers are not alone and encouraging the idea that there is no shame in seeking help both contribute significantly to bringing about changes in the current police culture.

In the workplace, peer-support is about co-workers, known as “peers,” who help one another through confidential discussions. In the general population, an employee’s personal problems can affect their job performance and, if left unchecked, these issues may lead to a decrease in their ability to function. The main objective of a peer-support program is to resolve employee and workplace problems before they escalate to crisis levels by providing an extra network of support in the workplace (Wallace, 2016). There is a consensus that the main goals of peer support are: to provide an empathic, listening ear; to provide low-level psychological intervention; to identify peers who may be at risk to themselves or others; and to facilitate a conduit for professional help. It is generally agreed upon that the goals of peer support do not relate solely to helping individuals recover from a traumatic or highly stressful incident; rather, peer-support functions to help maintain and promote psychological and physical health, and well-being more broadly (Creamer et al., 2012). Research into burnout and staff retention among health professionals has also identified the importance of peer support.

Peer-support programs that focus on operational, organizational and personal stressors in police organizations are relatively new. Traditionally, police services in Ontario were primarily reactive and the emphasis was on members who were involved with critical incidents (Marin, 2012). Therefore, it was necessary to examine peer-support programs in police organizations in the United States and Europe, as well as in other first-responder and non-first-responder professions, in order to obtain background information on these programs. For example, Burke et al. (2018) findings, suggest that peer-support can help empower people who are suffering from mental health issues and improve their self-efficacy.

Peer-support has been found to provide health professionals with positive validation, a sense of shared experience, knowledge and opportunity for reflective practice, stress and coping strategies, and enhanced self-confidence (Forster and Haiz, 2015). Peer-support can be defined as the true reciprocity and exploration of hope between individuals. For example, Armstrong et al. (1995) study of veterans found that peer-support does not necessarily involve sharing similar mental health experiences or traumas; in addition, it is a relationship that is based on empathetic listening and being compassionate human beings. Miyamoto and Sono (2012) suggest that one must consider how empathetic human relationships can be built and how one can challenge conventional attitudes about providing support. As such, it may be necessary to redefine the concepts of help and support. Furthermore, Heffren and Hausdorf’s (2016) study, which used the Distress Disclosure Index (DDI), revealed that police officers found it easier to speak to other officers when they were in a supportive environment. However, the literature review of police peer-support programs revealed that, thus far, no studies have examined how access to peer-support programs impacts officers’ overall mental health, or how established standards are important for the creation and maintenance of peer-support programs.

### Theoretical Framework

The suicides of nine police officers in Ontario in 2018 has sparked the provincial Minister of Community Safety and Correctional Services to initiate an inquest carried out by the Coroner of Ontario. Policy Feedback Theory (PFT) can be an important tool in bringing about change in police organizations, specifically attempts to change police culture in order to create an environment where police officers are encouraged to seek help for mental health issues. The importance of policy in effecting change has also been highlighted by Pierson (as cited by Wieble and Sabatier, 2018), who describes public policies as a “path dependent process whereby each step along a policy pathway makes it increasingly difficult to reverse course” (pp. 105–106).

Currently, police services in the province of Ontario are guided by Adequacy Standards, which set the direction for Chiefs of Police and provide the framework for what is required for policing in general. These standards are broken down into six categories: (a) Public Order, (b) Emergency Management, (c) Law Enforcement, (d) Victims Assistance, (e) Community Policing, and (e) Administration and Infrastructure. The goal of the Administrative Infrastructure category is for the government to create an Adequacy Standard related to the mental health and psychological wellness of police officers, including standards for peer-support programs and other mental health supports (Ministry of the Solicitor General, 2000).

PFT posits that the creation of official policy, including the attendant dedication of funding and resources, helps to organize, prioritize, and provide advantages for specific groups (Mettler and SoRelle, as cited by Wieble and Sabatier, 2018). In addition, PFT focuses on specific actors, networks, and ideas. Within the context of this study, the actors are police officers in the province of Ontario, the network is comprised of liaising between Ontario’s various mental health networks, and the idea is the creation and implementation of a realistic mental health initiative. As Cairney and Heikkila (2014) explain, when actors are present, policies are important to give rights to specific groups, whereas networks create opportunities for government agencies to mobilize support and to protect programs. As such, PFT forms the foundation for creating a shift in police culture based on organizational and provincial changes for police officers in the areas of mental health and peer- support. For example, Tucker (2015) found that officers who worked for agencies that had standing policies and procedures regarding officer wellness and other interventions for workplace stressors perceived a greater level of organizational support and were more likely to use mental health services.

### Purpose of the Study

This study was qualitative in nature, and it employed a phenomenological approach to achieve a better understanding of the impacts and workings of peer-support in a high-risk, high-stress organization. Data was acquired through one-on-one, face-to-face interviews using open-ended questions. The sample consisted of nine police peer-support team members who had served on the peer-support team for at least 2 years and had at least 10 years of policing experience. The purpose of this study was to obtain feedback from peer-support team members who have provided peer-support to members on a variety of issues, in order to gain insight into a number of related themes. These themes included, but were not limited to: (a) reducing the stigma associated with accessing mental health support; (b) whether talking to peers with similar experiences or general credibility helps to improve officers’ overall mental health; and (c) issues regarding a lack of a provincial standards.

### Participant Selection

Data was collected from a small, purposeful sample of nine sworn police officers who have served for at least 2 years on the York Regional Peer-Support Team. The participants had at least 10 years of police service and a variety of lived trauma and/or mental health experiences. All members who met the criteria were sent an email requesting their participation for a face-to-face interview. All ethical concerns related to recruitment materials and processes were addressed through the IRB, which granted its approval for this study on October 23, 2019.

An email was sent to all 36 police peers informing them of the opportunity to participate in a phenomenological doctoral study examining how their experiences providing peer-support has affected the overall mental health of officers, and peer team members’ perceptions regarding lack of a provincial standard. Twelve peer-support team members agreed to participate, but due to time, travel, and work schedules, face-to-face interviews were only conducted with nine police peers. To guard against potential concerns with confidentiality, the conference rooms selected were intentionally as far as possible from the offices of interviewees’ peers and senior officers. Due to geographical constraints, two participants were unable to meet at their own district, but agreed to meet at another district to be interviewed.

Before the interview began, informed consent was explained and the participant understood that their participation was voluntary. The participants were also informed that the interview would be audio recorded, and that notes would be taken. In order to keep the participant’s identity anonymous, their name, badge number, and current assignment were not used. Each interview was between 35 and 90 min. At the end of the interview, the participant was thanked for their time and reminded that they could contact the researcher if they had any questions or concerns regarding their interview.

### Demographics

Participants in the study had an average of 18 years of service, which included all major areas of the organization (e.g., operational, investigative, administration), and represented three different ranks. In addition, participants had at least 2 years of service as an official peer-support team member, and reported doing at least 10 h of peer-support (outside of their regular duties). In order to become a peer-support team member at the York Regional Police, members must have at least 5 year of service, be nominated by a peer, take part in a formal interview with two peer-team members and a clinical psychologist, and undergo a safeguard assessment to ensure suitability. The other main criterion is that the member must have lived (personal or professional) experience with a traumatic event. The participants interviewed for this study had either directly or indirectly experienced the following incidents: officer-involved shootings; the death of a civilian member while on duty; Special Investigation Unit (SIU) investigations; *Police Service Act (PSA)* investigations; discipline; coroner’s inquests; chief’s investigations; Office of the Independent Police Review Director (OIPRD) complaints; attendance to traumatic calls; living with members with addiction and mental health issues; physical injury in motor vehicle accidents that required accommodation at work; the deaths of a spouse and a brother; suicide of a family member; family member; and children with behavioral and developmental issues.

### Data Analysis

**FIGURE 1:**
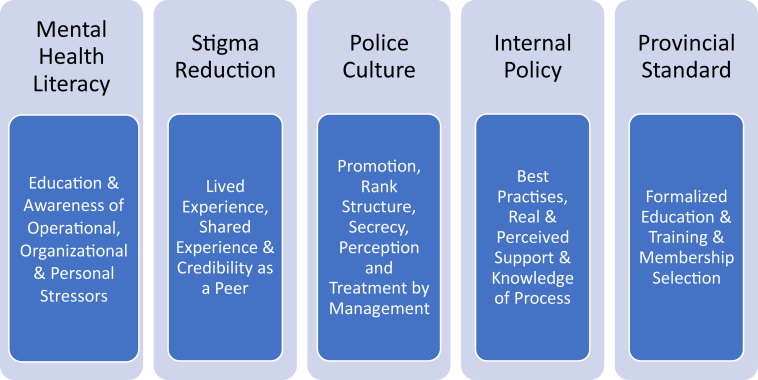
Emergent themes and sub-themes of peer-support effectiveness.

The data acquired from this phenomenological study was analyzed by first reviewing the interview transcripts for codes, significant categories, and themes, and then grouping the information according to the major themes and subthemes. It is through these themes and subthemes where a better understanding of how peer-support has actually helped police personnel and their families with a variety of different stressors was identified. Further, it came to light further suggestions to increase the legitimacy and risk management of these programs. In total, five themes were identified as listed in Figure 1: (a) mental health literacy; (b) stigma reduction; (c) effects of police culture; (d) the need for internal policy; and (e) the benefits of creating a provincial standard. The identified subthemes were as follows: for mental health literacy, participants cited education and awareness on operational, organizational, and personal support; for stigma reduction, participants cited lived experience, shared experience, and credibility; with respect to police culture, participants identified factors such as promotion, rank structure, secrecy, perception, and management; regarding the need for internal policy and best practices, participants mentioned mandatory training, real and perceived support, and knowledge of process; and participants identified formalized training and membership selection as significant subthemes with respect to provincial standards for peer-support programs in police organizations.

## Discussion

### Mental Health Literacy

Within a policing context, mental health literacy is defined as the ability to understand: the difference between mental health disorders and mental health issues; the importance of seeking treatment early and its role; the definition of stigma and how it relates to mental health; and how to develop competencies to improve one’s mental health (Kutcher et al., 2016). All participants agreed that mental health education, which includes resources and support for members after traumatic incidents, was minimal and support and resources for their families was non-existent prior to the establishment of the peer-support program.

Officer 1 noted, “for such a long time, mental health of members was completely under serviced.” This sentiment was echoed by Officer 5, who remarked that the police service was “great at taking care of public but [needed] to be better at taking care of our own.”

Eight of the participants reported that peer-support was more than just a conversation; to them, it was also a method of increasing members’ overall mental health literacy. They pointed out that mental health literacy among police officers had been improved through education about the differences between good, declining, and poor mental health; education on the differences between mental health issues and mental disorders; and the various sources of stress for police officers. This is important because there is a misconception that most police stress comes from attending traumatic incidents.

### Stigma Reduction

The stigma associated with mental health is still alive and well throughout society, but it is even more prevalent in police organizations. No one wants to come forward due to fears of being labeled, having their gun removed, and being transferred to a unit that is secretly referred to as “the land of broken toys,” suggested by the one of the peers. However, many of the participants reported that the creation of peer-support programs had been effective in decreasing the stigma surrounding mental health, and making officers feel more comfortable coming forward to seek help. The following is a selection of responses on this subject provided by the participants:

Officer 2: Peer-support has given officers the ability to speak and to be heard, judgment free.
Officer 4: There was no peer-support when I went through my shooting, when I meet with members I ask, what questions do you have for me, SIU or mental health related and after I get thanks for reaching out [.] knowing that someone has contacted me that has experience means a lot.
Officer 5: Due to stigma, personality in policing, a lot of people will go to a peer first before seeing a psychologist.
Officer 6: Peer-support is very positive, excellent progress on officers being more open about discussing issues they are facing.

Other forms of stigma reduction related to peer-support included the degree to which peer-support team members were seen as credible and trustworthy within the organization. For example, the idea of “peer-support” is strengthened by the peers’ perceived credibility due to their lived experience, which in turn makes them trustworthy. Indeed, the information gathered through the interviews is that one cannot provide high quality peer-support to a member who has been involved in a police shooting if they have not gone through the same experience themselves. On this matter, the respondents made the following remarks:

Officer 5: [A] key piece of peer-support is “credibility, trust” – [the] backbone of peer-support [is] lived experience – no rank.
Officer 1: Working in a high-risk unit gave me the opportunity to look at things in a different light [.] which lead me to help people I know.
Officer 9: [At the] time I started on the peer-support team [.] I noticed people would buy into [it] more because I was wearing a tactical uniform.
Officer 6: [The] main benefit of peer-support is the credibility of the members [.] [officers are] more likely to open up and be honest and share their issues, compared to being offered support by a complete stranger.

### Effects of Police Culture

The police culture has a big influence on how people behave within law enforcement organizations. Aspects of police culture suggested by the peers can include the decisions that are made, the quality of leadership, accountability, and whether members are being treated with fairness. The participants’ responses made it clear that organizational culture has a big influence on how members feel, think, and act. There was a consensus among the participants that police officers are more affected by “organizational stress” than they are by traumatic stress. For example, the promotional process appeared to be a huge source of stress that could affected members’ mental health if not channeled in the right ways. The following remarks reflect some of the respondents’ thoughts on the stress that can result from the promotions process:

Officer 4: Members report a crushing defeat of not getting promoted. [The] process is long and contributes to others’ jadedness, [and] moral injury that comes with not being promoted.
Officer 5: Members who are not promotable don’t feel value [.] [There are] high expectations on promotion and no education on promotion or self-reflection.
Officer 7: Promotion – big issues, takes up a ton of time, process is not fair, scores differ each year depending on who is marking, scores are manipulated, affects morale, not following code of ethics, affects officers’ mental health, [they] don’t want to come into work, don’t work as hard, a lot of time spent discussing the issue, general attitude is lowered, employees don’t feel valued.
Officer 8: [The] promotional process causes anxiety [and makes] people feel inadequate. Great police officers who are more than qualitied feel shook and question [their] sense of self. [They are] not sure of their abilities.
Officer 9: Promotions are a source of stress. [The] process is very subjective – [I’ve] spoken to many people about the flawed, huge holes [and how it] makes people feel like shit. [The] same material gets scored differently and [members] think they don’t have the skills and abilities to become a leader –[it’s a] huge hit to their self-confidence, motivation. [They feel like] their work has no purpose for advancement in their career.

Three participants also discussed how they had provided peer-support for a members who were being bullied in the workplace. This can include supervisors bullying members or co-workers with co-workers. As the respondents noted, regardless of whether the issue of bullying and/or harassment are perceived or real, it still represents a source of stress within the organization.

Officer 2: Stress from members is more than just the job. [It includes] everything from [the] promotional process, [to] bullying, [to] internal politics, [to] compassion fatigue.
Officer 9: I’ve had to peer-support two members that were being bullied [.] [I] gave them ideas of how to work around it, [and] gave feedback to help them through it.
Officer 7: I peer-supported a member who was bullied [.] I connected them with a mental health professional to give them tools [.] I later found out from the member [that they] had contemplated taking their life [.] [I was] told by the member that receiving help (peer and professional) had saved them.

### Need for Internal Policy

Police organizations are still considered as paramilitary organizations with a ranking structure and set policies and procedures. Policies are necessary because they clearly explain what is expected of the members, and they ensure that members are following protocol. Thus, there was no surprise that all participants agreed that an internal policy on peer-support is essential.

*Officer 1: Peer-support should be driven by the top down and should have a formal policy [.] mental health, use of force, prudent to not slough off someone who is having issues that needs their gun taken away –* laid out so everyone understands it – creation of a policy is to be in the members’ best interest.
Officer 4: Policy is a necessity [.] peer-support is signed on to by the Chief/organization and is now entrenched and we are buying into [it] – “gives it credence”[.] shows that it is important, supporting our people –[it is] seen as a place to go if there are questions and [that’s] hard because there is a lot of gray areas – defined wording sets a standard for the service and something we can fall back to.
Officer 5:[Policy] shows [the] organization is committed. [It] paints everyone with the same brush.
Officer 6: Internal policy would help – framework to what is close. The benefit would be consistency instead of negative. Peer-support engagement is driven by the members, but [it’s] good to have a policy [.] identifying what is required.
Officer 7: Each police service should have a policy in place so members can go and refer to it to assist them and what peer-support does.
Officer 8: Internal policy, support coming [from] within [.] stronger faith and commitment to the organization [.] send message that the Chief fully supports it.
Officer 9: Policy shows supervisors [which] courses of actions to take.

### Creation of a Provincial Standard

Police services in the province of Ontario are guided by Adequacy Standards, which create the framework for how police services operate and what they are mandated to have. Currently, there is no provincial standard for peer-support or any other mental health programs. The findings of this research revealed that not having a provincial standard can pose a certain level of risk, as peer-support in police organizations is much different than peer-support in the civilian world. Some of the issues identified by the participants included the fact that police carry firearms and they deal with victims of crime and trauma which makes them more prone to compassion fatigue and burnout. Furthermore, the respondents noted that police services are dynamic, which means there needs to be a set of best practices with respect to selecting and training peer-team members.

Officer 6: Each agency is dynamic, [but there are] common themes in every organization. Put in some best practices, allow those to guide legislation.
Officer 9: [The] ability for smaller services to have peer-support [.] [will reduce the] risk of people falling through the cracks.
Officer 5: Peer-support is good, but if police services are not following best practice or programs that work, [then it’s] not going to work.
Officer 3: YRP has learned the negatives and positives when providing peer-support [.] through a provincial standard able to share feedback and not make same mistakes.
Officer 2: Some agencies are way ahead where others have nothing [.] [It’s] not fair for police officers who do the same job[.] that one service has more than another, especially in the way of mental health and support.

As part of a provincial standard, participants identified education and the screening of peer-team members as criteria that should receive extra consideration when creating a provincial standard. For example, there is no set training course designated for peer-support for law enforcement. As a result, any police service in the province can implement any type of training (1 day to 2 weeks) offered by anyone (regardless of their qualifications) which is problematic. Also, screening and choosing peer team members is driven by the person running the team and very few police services psychologically screen their members for suitability prior to becoming a member.

Officer 8: A provincial standard [.] same process for vetting members [.] more stringent but finding out motives and intentions.
Officer 6: Not screening members can have a huge detriment – [it’s important] having officers [with] a natural aptitude to help others, credibility, being nominated, someone supports you.
Officer 5: As part of the standard, mandatory training should include suicide awareness, mental health first aid and The Working Mind for First Responders because of the common ground in language.

The Mental Health Commission of Canada has developed Peer-Support Guidelines and a National Standard for Psychological Health and Safety in the Workplace. Although both sets of guidelines provide a good starting framework, they are not inclusive of first responders. Police peer-support programs have an additional element of risk because members are deemed to be in “safety sensitive” positions. Therefore, the creation of a provincial standard for mental health support and resources that includes guidelines for peer-support in police organizations would support the PFT. The creation of a policy helps to organize, prioritize, and provide advantages for specific groups, and it demonstrates commitment and dedication and sets a pathway that makes it more difficult for organizations to look back (Mettler and SoRelle as cited by Wieble and Sabatier, 2018).

### Limitations

There are numerous limitations to this study. The data collected in the study was from one medium-sized police organization in Ontario, Canada. The use of just one police organization and a purposeful sampling does not provide an ability to generalize outside of the police organization to other police organizations. In addition, York Regional Police is known to have a robust and successful peer-support program with support from their Chief. This cannot be said about all peer-support programs in the province.

Lastly, there was an inherent bias as the researcher is a police officer and a member of the peer-support team. However, the bias was minimized by letting the participant tell “their experiences” and, allowing them to expand on their experiences without opinions, beliefs, feelings or self-knowledge by the researcher.

### Future Recommendations

Future research should include interviews with peer-team members at other, similar municipal police services throughout Ontario and/or with peer-team members serving on departments that already have an internal peer-support policy. Other research could also examine the overall impact of peer-support in other first-responder contexts, such as fire, paramedics, corrections and health care. Furthermore, this study was based on interviews with peer-support team members. Future research should explore the experiences of police officers and/or family members who have accessed peer- support.

## Conclusion

This purpose of this study was to gain information regarding the overall effectiveness of peer-support programs on police officers’ mental health, and to explore the implications of not having standardized peer-support policies. Peer-support programs have been in existence for decades in the United States, but have only recently gained in popularity in police services in Ontario, Canada. This is due to an increase in the number of police officers suffering from psychologically injuries and the unprecedented and alarming number of police suicides in 2018. This qualitative study revealed that peer-support team members viewed peer-support as being more than just a conversation. In their experience, peer-support was an indispensable tool for helping police officers learn about themselves, mental health, and the importance of seeking help early. In addition, the participants in this study strongly agreed that organizations need to implement internal policies regarding peer-support, which include the need to develop an adequacy standard for peer-support and mental health training. The findings of this research also revealed that peer-support team members provide the most support for “organizational stressors.” Examples of these stressors include the promotional process, police culture, and unsupportive supervisors. Incidents related to these stressors seem more prevalent and ongoing compared to traumatic incidents such as police shootings. This information can be very helpful for police organizations that are looking to incorporate new strategies or policies. The research also suggested that internal policy on peer-support would clearly establish the Chief’s, and the organization’s, commitment to mental health, as well as the role of peer-support team members.

Finally, it is imperative to create a provincial mental health standard, or, at the very least, to provide guidelines for peer-support in police organizations in Ontario, Canada similar to the International Association of Chiefs of Police (IACP) peer-support guidelines to ensure best practices and a level of risk management.

## Data Availability Statement

All datasets generated for this study are included in the article/supplementary material, further inquiries can be directed to the corresponding author.

## Ethics Statement

The studies involving human participants were reviewed and approved by the Institutional Review Board at the Walden University. The patients/participants provided their written informed consent to participate in this study.

## Author Contributions

The author confirms being the sole contributor of this work and has approved it for publication.

## Conflict of Interest

The author declares that the research was conducted in the absence of any commercial or financial relationships that could be construed as a potential conflict of interest.
